# Photobiomodulation and Sericin in the Treatment of Second‐Degree Burns Induced in Wistar Rats

**DOI:** 10.1111/wrr.70135

**Published:** 2026-02-23

**Authors:** Lilian de Araújo Pradal, Mustafa Munir Mustafa Dahleh, Marina Prigol, Gustavo Petri Guerra, Celeste da Rocha Paiva, Thaís Caroline Schnaufer, Lucinéia de Fátima Chasko Ribeiro, Rose Meire Costa, Gladson Ricardo Flor Bertolini

**Affiliations:** ^1^ Universidade Estadual do Oeste do Paraná – UNIOESTE Cascavel Brazil; ^2^ Universidade Federal do Pampa – UNIPAMPA Itaqui Brazil

**Keywords:** burns, photobiomodulation, sericin, wound

## Abstract

This study investigated the isolated and combined effects of sericin and low‐level laser photobiomodulation (PBM) on the healing of experimentally induced second‐degree burns in male Wistar rats. Sericin has previously been associated with enhanced fibroblast migration and improved reepithelialization, while PBM has shown anti‐inflammatory potential. To assess their therapeutic value, 60 rats were randomised into six groups: untreated control (CON), silver sulfadiazine (SUL), sericin cream (SER), PBM alone, SUL + PBM and SER + PBM. Burn injuries were produced using a heated metal instrument applied for 3 s, and animals were euthanized 14 days later. Macroscopic photography, photothermography and histomorphometric analyses were performed. Statistical tests using generalised linear models were set at *p* = 0.05. A significant temperature difference between initial (AV0) and follow‐up (AV1) evaluations was observed, although granulation tissue formation did not differ across groups. Collagen analysis revealed that SER, PBM, SUL + PBM and SER + PBM groups presented higher collagen scores than controls (*p* < 0.05). The SER + PBM treatment was particularly effective, showing the greatest proportion of type I collagen and statistical superiority over the other interventions (*p* < 0.05). Molecular evaluations demonstrated reduced levels of tumour necrosis factor alpha, thiobarbituric acid reactive substances and catalase, especially in groups receiving PBM in combination with either sericin or sulfadiazine, suggesting a decrease in oxidative stress. Overall, the results indicate that both sericin and PBM positively influence tissue repair, with combined therapy—particularly SER + PBM—yielding enhanced collagen deposition and biochemical markers consistent with improved healing. Further studies are warranted to clarify the underlying biological mechanisms driving these outcomes.

## 1 Introduction

One of the causes of skin lesions is burn injuries [1], is characterised as an inflammatory post‐traumatic pathological process, accompanied by local and systemic effects, leading to an intense inflammatory response, structural and functional tissue damage [2, 3]. These lesions are classified according to depth, tissues affected and severity. In first‐degree burns, only the epidermis is affected, with local erythema and pain to the touch. Second‐degree burns affect the epidermis and dermis, are painful and sensitive to touch. In third‐degree injuries, the epidermis, dermis and hypodermis are affected and in fourth‐degree injuries the destruction reaches deeper tissues and is painless due to the destruction of free nerve endings [1, 4, 5].

In terms of pathophysiology, immediately following trauma, the initial phase of the inflammatory process involves the recruitment of neutrophils and monocytes to the affected site. This recruitment is mediated by vasodilation and the extravasation of fluids into the adjacent interstitium, subsequently eliciting an immune response that is sustained by macrophages and chemocytokines, such as tumour necrosis factor alpha (TNF‐α), interleukin‐1 (IL‐1) and interleukin‐6 (IL‐6), central elements of this process [6, 7]. The inflammatory process also stimulates cellular metabolism at the burn site, leading to increased production of reactive oxygen species (ROS) such as superoxide (O_2_^−^), hydroxyl (OH^−^) and hydrogen peroxide (H_2_O_2_). This inflammatory response becomes systemic, and TNF‐α starts to induce cell apoptosis [8].

The definition of best treatment for such injuries is determined by a set of variables, such as the depth, extent, location and cause of the burn [9]. The utilisation of intravenous antibiotic therapy presents limitations, including challenges in achieving adequate drug penetration to the wound bed and potential systemic toxicity to the patient. This necessitates the use of topical agents, predominantly antimicrobials, such as 1% silver sulfadiazine. Silver sulfadiazine is widely employed due to its ease of application, low cost and capacity to modulate pro‐inflammatory cytokines [5, 6, 7, 9, 10].

Sericin, a proteinaceous material produced by *Bombyx mori* , the silkworm larva, has been investigated as an alternative in the management of cutaneous lesions. This is attributed to its amino acid composition, which exhibits similarity to that of the stratum corneum, as well as its biocompatibility and low toxicity profile [11]. Studies have indicated that sericin enhances fibroblast migration [12], increases the hydroxyproline content in the stratum corneum, decreases skin impedance and induces collagen production [13, 14], without the activation of pro‐inflammatory cytokines [15].

Another widely discussed modality of therapy is photobiomodulation (PBM), which can be performed with both low‐power lasers (LBP) and LEDs, and according to Mosca and colleagues [16], is effective in stimulating wound healing, decreasing the inflammatory process and pain by reducing vascular permeability and edema. The main target of the visible radiation emitted by the equipment is cytochrome C oxidase (COX), located in the mitochondria [16, 17].

However, despite the aforementioned evidence, the literature exhibits a paucity of data regarding the combined application of sericin and low‐level laser therapy in the management of burns. Thus, it is believed that sericin in topical cream form may improve the morphophysiological and morphometric aspects of skin in experimentally induced burns. Therefore, this research aims to test this hypothesis and develop a treatment protocol for second‐degree burns, since these are notably painful and to approximate the clinical reality of patients affected by these injuries, with rats being a widely used experimental animal due to their similarity to human physiological responses, ultimately improving the rehabilitation process and providing healthcare professionals with novel therapeutic resources for patient treatment.

## 2 Methods

### 2.1 Animals and Experimental Groups

The study was approved by Unioeste's Animal Research Ethics Committee under protocol 16‐21/2021. The sample group consisted of 60 male Wistar rats, weighing approximately 350 g, obtained from the Central Animal Facility of the State University of Western Paraná—UNIOESTE, which were kept in standard polypropylene plastic boxes, with access to water and food ad libitum, a controlled temperature of 21°C ± 1°C and a 12‐h light/dark photoperiod.

### 2.2 Experimental Design

The 60 animals were randomised into six groups with 10 animals each: Control injury group (CON)—animals subjected to injury without any type of treatment; sulfadiazine injury group (SUL)—animals subjected to burn injury and treated with silver sulfadiazine ointment; sericin injury group (SER)—animals subjected to burn injury and treated with sericin cream; PBM injury group—animals subjected to injury and treated only with laser; LBP standard ointment injury group (SUL + PBM)—animals subjected to injury and treated with silver sulfadiazine standard ointment and laser; sericin laser injury group (SER + PBM)—animals subjected to injury and treated with sericin cream and laser.

### 2.3 Experimental Model of Second Degree Burns

The burn induction was performed in accordance with protocols established by Chiarotto et al. [18], with adaptations. The animals were anaesthetised via intraperitoneal injection with xylazine (50 mg/kg) and ketamine hydrochloride (75 mg/kg). Subsequently, the interscapular region of each animal was trichotomized, and the area was aseptically prepared with 70% alcohol. The instrument employed consisted of a rectangular wooden block with an attached metal plate, possessing a total weight of 100 g. The metal plate had a surface area of 1 cm^2^, inducing an initial lesion of equivalent dimensions. The burn induction instrument was heated using a Bunsen burner for 30 s and then maintained in contact with the trichotomized skin of the animals for 3 s to create a second‐degree burn, as previously established in a pilot study, characterised by complete epidermal destruction and partial dermal destruction.

Following the procedure, once the animals had regained consciousness, they were administered oral dipyrone at a dosage of 50 mg/kg, as needed, whenever they exhibited signs of nociception [19, 20].

Mechanical debridement of the eschar was deliberately avoided; instead, spontaneous separation through natural sloughing or liquefaction was permitted to occur, thereby preventing traction injury to the granulation tissue and reducing potential pain or discomfort in the experimental subjects.

### 2.4 Silver Sulfadiazine Treatment Protocol

The treatment protocol for the group treated with silver SUL also began immediately after the burn was induced.

The animals were treated with a 1% silver sulfadiazine ointment. The ointment was topically applied to the wound bed in a quantity sufficient to cover the entire lesion. Following treatment application, an occlusive dressing was applied and subsequently changed every 24 h, as for every single animal, allocated in any group. The dressing consisted of gauze, secured with adhesive tape to achieve occlusion, after which each animal was fitted with a vest to prevent dressing removal. These vests were constructed from an unbleached cotton fabric with a thread count of 180 and featured a Velcro front closure, adhering to the garment design proposed by Borges [21] and Borges et al. [22] with adaptations. All the animals in all the groups wore this outfit.

The ointment used was commercial Dermazine Silvestre Labs, formulated with cetostearyl alcohol, stearyl ether, ethoxylated oleyl alcohol, methylparaben, propylparaben, petroleum jelly, propylene glycol, deionised water and 1% micronised silver sulphadiazine.

### 2.5 Sericin Treatment Protocol

The treatment protocol for groups a with sericin (SER and SER + PBM) began immediately after burn induction, with 4% sericin cream, following the principle of minimum effective dose [23]. The cream was topically applied to the burn site in a quantity sufficient to cover the entire lesion. Following treatment application, an occlusive dressing was applied, consisting of gauze secured with adhesive tape. The dressing for each animal was changed every 24 h.

The cream was formulated with white petroleum jelly, mineral oil, lanolin, glycerin, bisabolol, triethanolamine stearate, propylparaben, methylparaben and 4% sericin [12, 15]. The cream was formulated and prepared by the Pharmacotechnics and Cosmetology Laboratory at UNIOESTE—Cascavel.

The process of extracting sericin from the *B. mori* cocoon and freeze‐drying it to produce the cream took place at the Laboratory of Microorganism Biochemistry at UNIOESTE—Cascavel, according to the procedure proposed by Debastiane et al. [24].

### 2.6 PBM Treatment Protocol

For the groups that underwent PBM (PBM, SUL + PBM and SER + PBM) the equipment of choice was laserpulse (Ibramed, 30 mW, 670 nm), and point therapy, at four points (vertices of the wound), with an energy density of 4 J/cm^2^ at each point, power of 30 mW, irradiance of 1.07 W/cm^2^, for 10 s at each vertex, that is, 1.2 J of total energy delivered. PBM therapy began 24 h after the burn was induced and was carried out every 48 h, according to the protocol of Silveira et al. [25] and Lamaro‐Cardoso et al. [26].

Dressing changes took place in the same way for all the animals, each receiving their respective treatment, as previously reported.

### 2.7 Macroscopic Evaluation and Epithelialization

Macroscopic assessments of lesions were carried out starting on day 1, immediately after lesion induction and repeated every 24 h.

The animals were manually restrained by the investigator to expose the injured area. A 12‐megapixel camera with an aperture of f/2.2 was employed. The camera was positioned perpendicularly above each animal, 20 cm from the supporting surface and affixed to a tripod. The wounds were photographed to quantify the reduction in the burn area over time. The acquired images were transferred to a computer and subsequently analysed using Image‐Pro Plus 6.0 software via planimetry to assess the evolution of the lesion area.

### 2.8 Temperature Evaluation

A thermographic camera (Flir C5—Teledyne Flir, Santa Barbara, USA) was utilised to assess the temperature of the injured area. These data were recorded immediately following the burn induction and subsequently repeated every 48 h, both prior to treatment application and after wound dressing cleaning. Thermographic images were also acquired prior to injury induction to serve as a control. Temperature measurements were obtained at the centre of the lesion and laterally to the outer margin of the burn [27, 28] and its analysis was carried out using the Flir Thermal Studio 2.021 software.

### 2.9 Histological Processing of the Dermis Light Microscopy

On the 7th and 14th days, the animals were euthanized by deep anaesthesia with ketamine hydrochloride (95 mg/Kg) and xylazine hydrochloride (45 mg/Kg) via intraperitoneal injection. After checking the animal's state of consciousness (observed by the absence of motor response to the pinching of the tail and interdigital folds), samples were taken from the intact and burn‐injured skin.

After being collected, the animals' skin samples were washed with 70% alcohol and fixed in 10% formalin. The pieces were then dehydrated in an increasing series of alcohols (70%, 80%, 90%, 100% I, 100% II and 100% III), diaphanized using xylol alcohol I, II and III and embedded in histological paraffin.

Subsequently, microtomy was performed by obtaining sequential sagittal sections of 5 μm on an Olympus microtome (R CUT 4055 Tokyo, Japan) to obtain histological slides of the tissue. The slides were stained with haematoxylin and eosin (HE) for histomorphometric analysis of epidermis and dermis, and picrosirius red for collagen fibre analysis. After staining, the slides were analysed and photomicrographed using a light microscope at 40× objective, photographing three samples per animal at the transition line from burnt to healthy skin (Olympus DP71, Tokyo, Japan) for HE, and a polarised light microscope (Zeiss Axio Scope A1, Zeiss Industrial Metrology, Germany), photographing three samples per animal at the transition line using a 40× objective for the slides stained with picrosirius.

**FIGURE 1:**
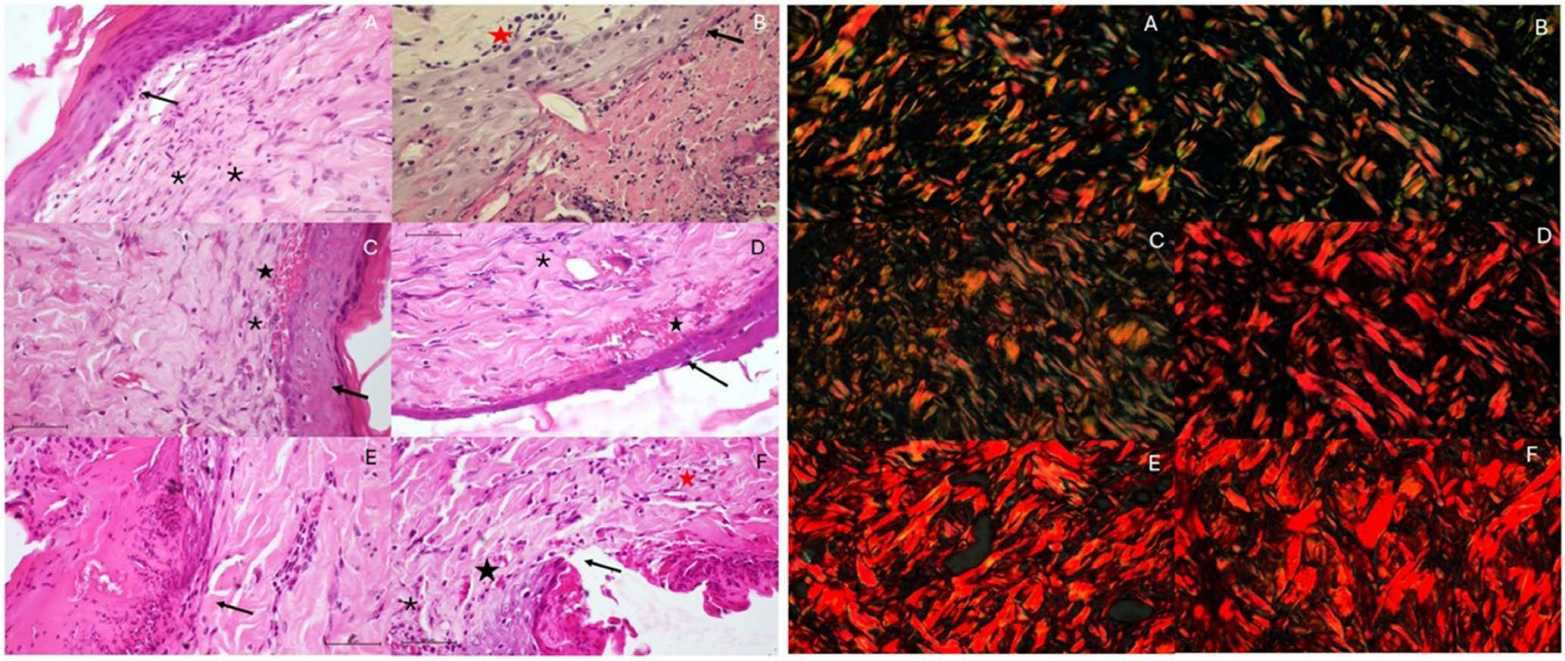
On the right, photomicrograph of the transition line from burn to intact skin, in a sagittal section stained in HE. (A) CON—control; (B) SUL—sulfadiazine; (C) SER—sericin; (D) PBM—laser; (E) SUL + PBM—sulfadiazine + laser; (F) SER + PBM—sericin + laser. Shown are the transition lines (black arrows), blood vessels (black stars), fibroblasts (asterisks) and defence cells (red stars). On the left, photomicrograph of the transition line from burn to healthy skin, in a sagittal section stained with picrosirius. (A) CON—control; (B) SUL—sulfadiazine; (C) SER—sericin; (D) PBM—laser; (E) SUL + PBM—sulfadiazine + laser; (F) SER + PBM—sericin + laser. The bar shown in the photomicrographs represents a distance of 100 μm.

To analyse the HE‐stained slides, a semi‐quantitative analysis was carried out considering values from 0 to 4 according to the scale in Figure 1, following the score used by authors who also evaluated experimentally‐induced burns [29, 30, 31, 32] and proposed by Kumar et al. [33]. The scale proposed by Robbins and Cottran was also used to analyse collagen deposition [33], through semi‐quantitative analysis. To this end, the percentage of pixels corresponding to the staining of each collagen fibre type in photomicrographs was quantified. Under polarised light microscopy, type I collagen fibres exhibited red/yellow or orange birefringence, while type III collagen fibres displayed green birefringence [30, 34]. The percentage of each colour expressed in pixels was calculated using the GIMP 2.10.34 software.

### 2.10 Inflammatory Profile Analysis

Samples of intact and injured skin from each animal were used to analyse the inflammatory profile. The samples were collected after euthanasia, placed in cryotubes and then frozen in an ultra freezer at −86°C (Indrel Ultra Freezer −86°C, Indrel Scientific Londrina, Paraná). TNF‐α was analysed using an ELISA kit, following the manufacturer's instructions, that is, 100 μL of diluted or standard samples of recombinant mouse TNF‐α were added to a 96‐well plate pre‐coated with anti‐TNF‐α and left for 2 h at room temperature (RT). After washing, 100 μL of biotin‐conjugated antibody was added and left for 1 h in TA. After another wash, 100 μL of streptavidin‐peroxidase (HRP) was added and left for 30 min in TA, followed by additional washes. The reaction was then revealed by adding 100 μL of stabilised chromogen, incubated for 30 min at TA and then the reaction was stopped by adding a stop solution. Readings were taken on an ELISA microplate reader (VERSA Max, Molecular Devices, California, USA) at a wavelength of 450 nm. The concentrations were calculated from a standard curve prepared with recombinant TNF‐α. The detection limit was 11.7–750 pg/mL for TNF‐α [35].

### 2.11 Analysis of the Antioxidant System

Samples of intact and damaged skin from each animal were used to assess the antioxidant system. An aliquot was taken from these samples, stored in microtubes with Tris–HCL buffer, with the pH adjusted to 7.4, and then stored again at −80°C.

The crude samples were then centrifuged in a microcentrifuge at 12,000 rpm (13,680 G) at 4°C for 12 min. The supernatant was pipetted off and placed in new microtubes. Protein determination followed the Bradford method. Once the protein concentrations of the samples had been determined, they were normalised to 1 mg of protein per 1 mL of sample.

The enzymes superoxide dismutase (SOD) and catalase (CAT) were analysed, as well as reactive species and levels of malondialdehyde (aldehyde product derived from lipid peroxidation [thiobarbituric acid reactive substances, TBARS]). For SOD, the analysis followed the method proposed by Marklund and Marklund [36]. The results were expressed as U of SOD/mg of protein. Catalase activity was measured using the rate of decrease in the absorbance of hydrogen peroxide at 240 nm [25], lipid peroxidation was quantified using TBARS and expressed in malondialdehyde equivalents (MDA). The absorbance was measured at 535 nm using a UV–Visible spectrophotometer (Bio‐Rad), and the values were expressed as nmol MDA/mg protein [37].

### 2.12 Statistical Analysis

Statistical analysis was carried out using SPSS 20.0 software and GraphPad Prism 8.4.3. Data were expressed as mean ± standard deviation. The variables were analysed using Generalised Linear Models, with LSD post‐test. The difference was considered significant when *p* ≤ 0.05, with a 95% confidence interval.

## 3 Results

### 3.1 Macroscopic Assessment of the Lesion Area

**FIGURE 2:**
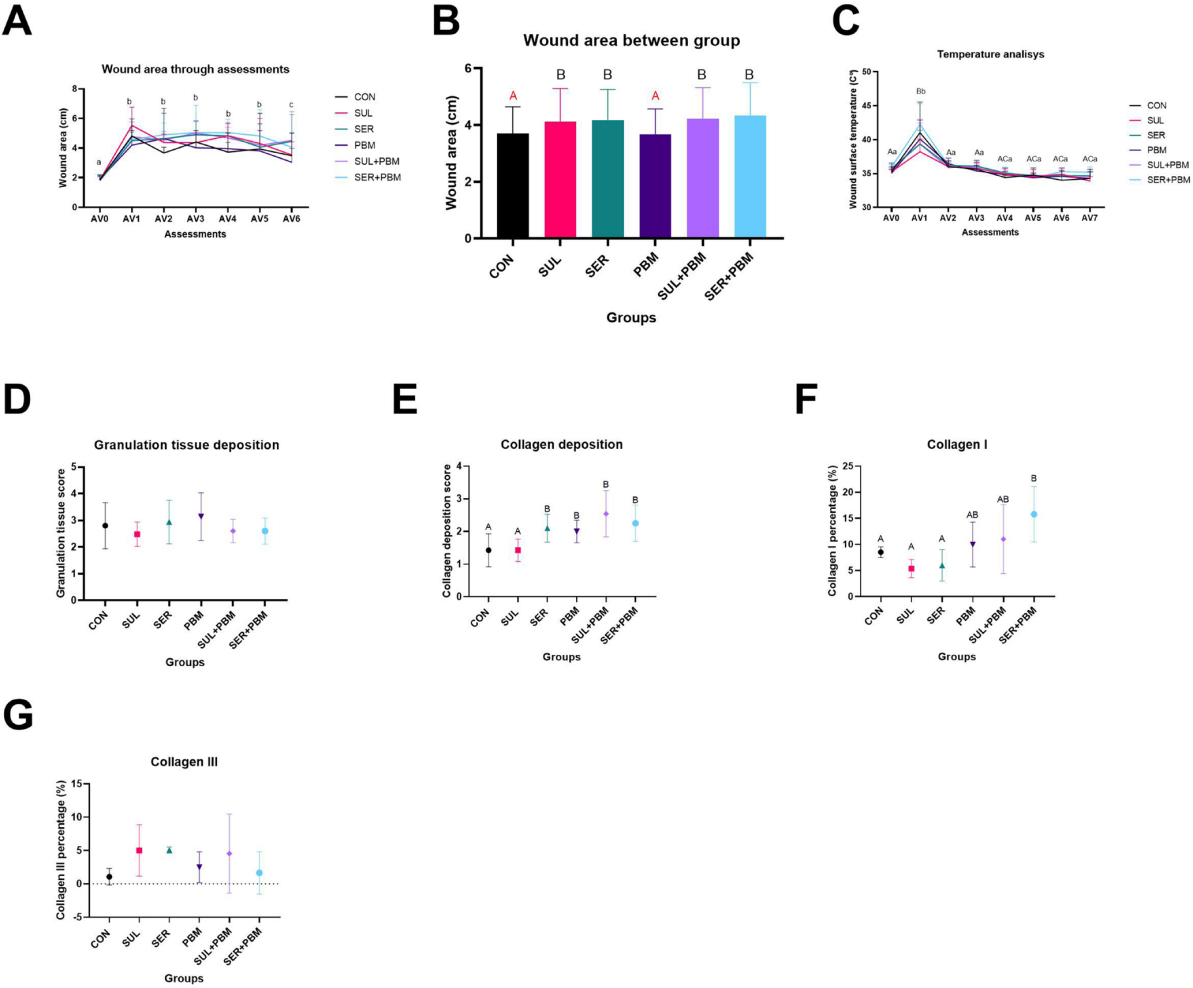
Results of the lesion area analysis (A and B). Graphs showing the evolution of the lesion area. (A) Evaluation of the area between the groups. (B) Evaluation of the lesion area over the time of the experiment. CON, control; PBM—laser; SER + PBM—sericin + laser; SER, sericin; SUL + PBM—sulfadiazine + laser; SUL, sulfadiazine. (C) Results of lesion surface temperature analysis. The graph shows the temperature evolution over the time of the experiment between the groups. (D) Graph showing the position of the groups on the granulation tissue formation scale. (E) Graph showing the position of the groups on the collagen deposition scale. (F) Percentage of type I collagen. (G) Percentage of type III collagen. Results expressed as mean ± standard deviation, indicated by the bars above the columns and rows. Different letters represent statistically different values. Lowercase letters, comparison within the same group; uppercase letters, comparison between groups; equal letters show statistical similarity.

No factor interactions were observed in the analyses carried out; however, the CON and PBM groups were similar to each other (*p* = 0.798), but there was a statistical difference between these two groups and the others (*p* < 0.05), which in turn were statistically similar. As for the assessments over time, there was a statistical difference between AV0 and all the others (*p* < 0.01), AV1–AV5 showed no difference (*p* > 0.05), while the last assessment, AV6, was different from all the others (*p* < 0.05), as shown in Figure 2.

### 3.2 Temperature Assessment

Between groups, there was a difference between AV1 and the other assessments (*p* < 0.05) while the others were similar to the initial one, AV0 (*p* > 0.05). There was no statistical difference in the analysis between the groups, but there was an interaction between the groups and the evaluations over the time of the experiment, since after inducing the injury, in AV1, all the groups showed an increase in skin surface temperature (*p* < 0.01), after 48 h in AV2 and after 96 h in AV3, they were similar to AV0 (*p* > 0.05), in the following evaluations, the groups were still the same as AV0 (*p* > 0.05), but different from AV2 and AV3 (*p* < 0.05), as shown in Figure 2A.

### 3.3 Semi‐Quantitative Histomorphological Assessment

When analysing the granulation tissue deposition score, there was also no statistical difference between the groups (*p* > 0.05), as shown in Figure 1. Just below, in Figure 1, it is possible to see the transition line between burn and intact skin again, but in some samples, it is already possible to see re‐epithelialization. In some groups there is still a pyogenic membrane (CON and SUL), migration of less abundant defence cells (SUL) and fibroblasts in large quantities in some samples (SER + PBM).

Regarding the collagen deposition score, the CON and SUL groups were statistically similar (*p* = 1.0), but different from the other groups (*p* < 0.05) which showed greater collagen deposition, while SER, PBM, SUL + PBM and SER + PBM were the same (*p* > 0.05), as shown in Figure 2A.

As for the percentage of type I collagen, the CON, SUL and SER groups were similar (*p* > 0.05), SER + PBM showed a higher percentage of collagen I deposits and was statistically different from CON, SUL and SER (*p* < 0.03). The PBM and SUL + PBM groups were the same (*p* = 0.693), similar to CON and SER + PBM (*p* > 0.05) but different from SER (*p* < 0.05). An illustration is shown in Figure 2B.

Concerning collagen III deposition, there was no statistical difference between any group (*p* > 0.05), as shown in Figure 2C. Figure 1 showing the transition line from burn to healthy skin in polarised light photomicrographs.

**FIGURE 3:**
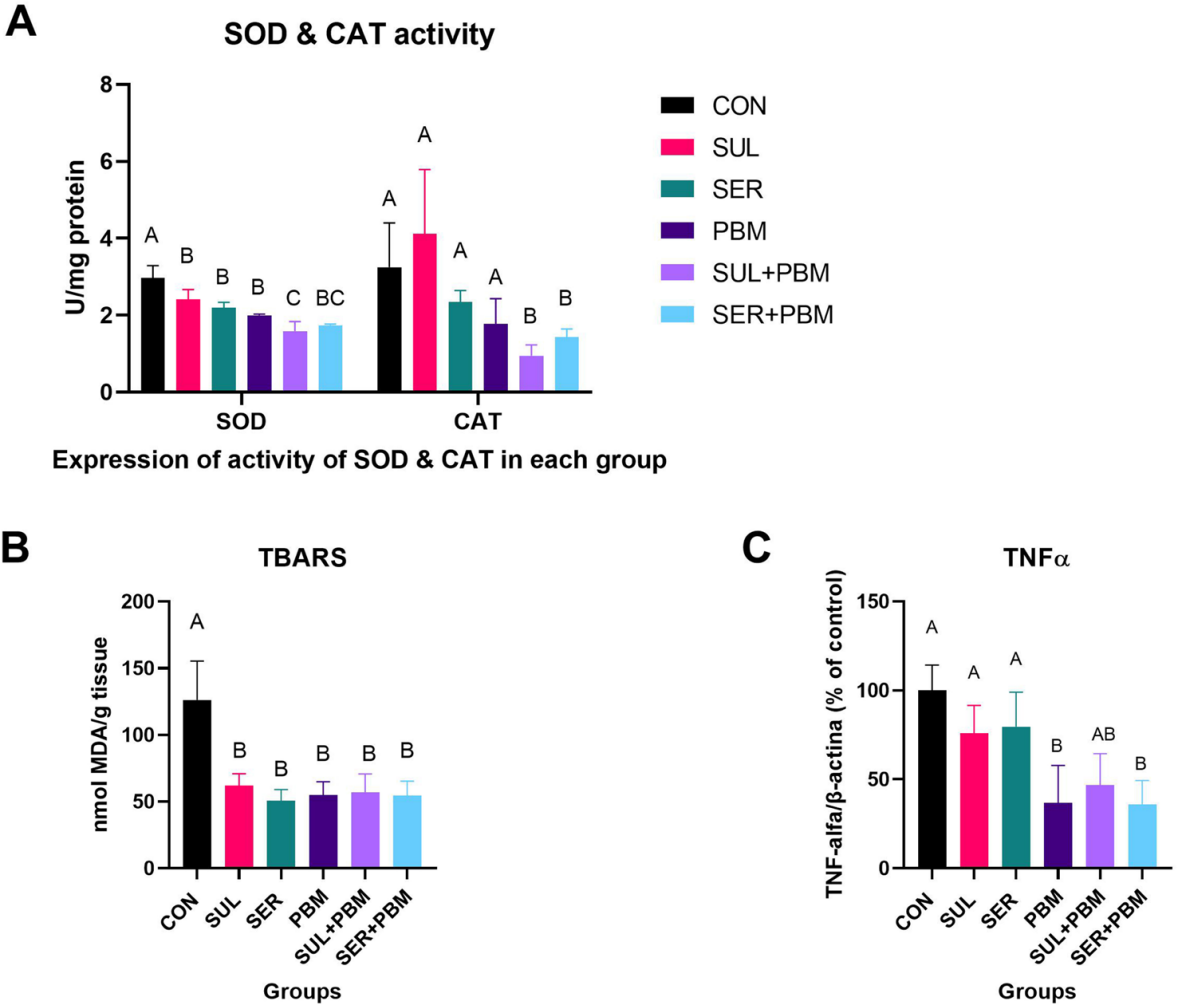
Graphical results of the analyses of SOD and CAT activity (A), TBARS (B) and TNFα (C). CON, control; PBM—laser; SER + PBM—sericin + laser; SER, sericin; SUL + PBM—sulfadiazine + laser; SUL, sulfadiazine. Results expressed as mean ± standard deviation, indicated by the bars above the symbols for each group. Equal letters show statistical similarity.

Pertaining to SOD expression, CON was statistically different from the other groups (*p* < 0.05), SUL, SER and PBM were the same (*p* > 0.05), and SUL + PBM and SER + PBM were the same (*p* = 0.46) and different from the control (*p* < 0.001). In the analysis of CAT expression, there was no difference between CON, SUL, SER and PBM (*p* > 0.05), these groups being statistically different from SUL + PBM and SER + PBM (*p* < 0.01), the latter being similar with a *p*‐value of 0.9. As for TBARS, there was a statistical difference only between the CON control and all the other groups (*p* < 0.01), with the others all being the same (*p* = 1). In the TNF‐α analysis, CON, SUL and SER were the same (*p* > 0.05), while PBM, SUL + PBM and SER + PBM were similar (*p* < 0.05) but different from the first 3 (*p* < 0.05), as illustrated in Figure 3.

## 4 Discussion

Considering the architecture of the skin, burns represent one of the most debilitating traumas, leading to the destruction of the affected tissues [3], the resolution of these wounds involves dynamic and temporally overlapping phases. Immediately following trauma, haemostasis is initiated via local vasoconstriction, platelet activation and aggregation and the release of various factors by platelets, keratinocytes, macrophages and fibroblasts. These processes culminate in the formation of a clot, thereby transitioning to the subsequent inflammatory phase [38].

This occurs naturally in order to degrade necrotic tissue and initiate the cascade of signals needed to repair the injury. Cells such as monocytes, macrophages and neutrophils are recruited, with neutrophils and macrophages releasing cytokines and chemokines, consisting of IL‐1β, IL‐8 and TNFα, responsible for the release of IL‐6, and growth factors such as TGFβ, essential for the recruitment of fibroblasts, which secrete insulin‐like growth factor (IGF) and vascular‐endothelial growth factor (VEGF) [4, 38, 39].

With regard to thermography, a potential indicator of active inflammatory processes [40], statistically significant differences were found only at the moment immediately after the induction of the experimental burn, but no difference between the control and treated groups, possibly due to factors related to the animals' ability to thermoregulate or the small size of the injured body area [41].

Although no significant inter‐group differences were observed in wound size, an observation consistent with a prior study [42], the extracellular matrix deposited exhibited a richer constitution of type I and III collagen in all treated groups without statistical significance. This finding aligns with the results reported by Gomes et al. [43], where animals treated with PBM and sulfadiazine demonstrated comparable healing outcomes. However, the G6 group displayed significant collagen deposition, potentially associated with an accelerated lesion resolution process in this group. This acceleration may be attributed to the synergistic effect between PBM and the fibroblast stimulation capacity of sericin, possibly mediated by sericin binding to receptors and the subsequent activation of signalling pathways such as mitogen‐activated protein kinase/extracellular signal‐regulated kinase (MAPK/ERK) and transforming growth factor beta/small mothers against decapentaplegic (TGF‐β/Smad). These pathways are known to regulate the expression of genes related to the extracellular matrix, including type I and III collagen genes, as discussed in previous literature [14, 15, 44].

During the proliferative phase, fibroblasts and keratinocytes are recruited and activated at the wound site. This phase is characterised by the replacement of the provisional matrix with granulation tissue, the formation of a definitive extracellular matrix, angiogenesis and epithelialization. Subsequently, fibroblasts differentiate into myofibroblasts, which are involved in extracellular matrix remodelling [4, 38]. Overlapping, the remodelling phase begins, in which the granulation tissue matures and the scar is formed, as collagen and elastin are deposited and readjusted, and the extracellular matrix, formed by myofibroblasts, is remodelled under the influence of metalloprotease inhibitors (TIMP), which leads to increased tensile strength [6, 38].

As for the expression of TNFα in the analysed tissues, this study demonstrated that the experimental groups receiving combined treatments (PBM, SUL + PBM and SER + PBM) exhibited a reduced concentration of this cytokine. This observation corroborates the findings of other studies [45, 46], although those authors solely evaluated PBM as a treatment, and not its combination with silver sulfadiazine and sericin. These results suggest that the combined treatments promoted an acceleration of the inflammatory process compared to the control group. This inference is supported by the work of Rowman et al. [6], which indicates that higher levels of TNFα are associated with the initial phases of wound healing, including burns.

It is through the absorption of red or NIR light by the mitochondrial chromophores contained in the respiratory chain that the laser exerts its therapeutic benefits [16]. Irradiation showed inhibitory effects on the inflammatory markers prostaglandin‐2, TNFα and IL1β, stimulation of collagen production, activation of fibroblasts and on the expression of metalloproteinases. Thus, PBM alters, increasing the cellular redox state, which induces the activation of intracellular signals, altering transcription factors that promote cell proliferation and tissue repair [26, 47]. The use of PBM as a therapy also acts to modulate the activity of M1 and M2 macrophages through its influence on intracellular signalling, these macrophages being one of the main cells responsible for the release of TNFα [48].

As for the sulfadizine‐treated group, previous studies also endorse the above observation, showing that silver sulfadizine not only has antimicrobial properties, but can also modulate the inflammatory response [10], through its interaction with proteins and enzymes within immune cells, especially macrophages, affecting signalling pathways crucial for the transcription of pro‐inflammatory genes, such as TNFα, involving the modulation of transcription factors such as NF‐κβ, which plays a central role in regulating TNFα expression [49]. This would explain why the application of sulfadizine to burns reduced the expression of TNFα, leading to more effective healing of these lesions [50].

As for sericin, in addition to what was observed in this study, it has been shown to inhibit the activation of nuclear factor kappa B (NF‐κB) [51], a key protein in the regulation of the inflammatory response. NF‐κB, when activated, induces the transcription of pro‐inflammatory genes, including TNF‐α, so by blocking this pathway, TNF‐α production is reduced, a situation that may also be related to mitogen‐activated protein kinases (MAPK), which play a crucial role in the production of cytokines such as TNF‐α, because as some authors suggest, sericin modulates these pathways in down regulation, thus leading to lower TNF‐α expression [52, 53].

In addition to the modulation of the inflammatory process found in this research, TBARS, which quantify the concentration of MDA and other secondary products of the degradation of polyunsaturated lipids by the action of free radicals, triggered by an exacerbated production of ROS, such as superoxide (O_2_^−^), hydrogen peroxide (H_2_O_2_) and the hydroxyl radical (OH^−^) [8, 54], in the present study we found evidence that all the treatments were effective in reducing the initial oxidative damage of the burn, possibly through different mechanisms, such as the direct antioxidant action of sericin [15], modulation of the inflammatory response by sulfadiazine [50] and the PBM [30], since this marker remained elevated only in the untreated group.

Considering, therefore, the results found in this study, of the modulation of the inflammatory process through the reduction of inflammation by the downregulation of TNF and TBARS, which was more evident in general in the groups that received the combined treatments (PBM, SUL + PBM and SER + PBM), the results found suggest that the treatments used in this research had a beneficial effect on the resolution of the lesions.

In burns, the initial ischemia caused by vasoconstriction over the period of haemostasis phase and the subsequent reperfusion throughout the inflammatory phase is one of the factors that lead to local oxidative stress, which is characterised by an imbalance between ROS and the antioxidant defence system [8]. Pro‐inflammatory cellular activity, especially of neutrophils and the release of cytokines, as discussed above, also contribute to the formation of ROS and NROS, which eventually lead to lipoperoxidation, which in turn contributes to greater tissue degeneration at the site of injury, due to the increased concentration of MDAs since cell membranes are rich in polyunsaturated fatty acids, a vulnerable target for ROS [2, 38].

Thus, we found that there was a gradual reduction in the concentration of enzymes of the antioxidant system, SOD and CAT and in the evaluation of both enzymes, the most significant reduction in their expression occurred in groups SUL + PBM and SER + PBM, possibly because these groups, due to the combination of treatments, were led to an early maturation of the injured tissue, already transitioning from the proliferation phase to wound maturation [10, 51], which could also be related to the findings regarding the extracellular matrix.

## 5 Conclusions

In short, the results of this research indicate that the treatments applied, especially the combinations (SUL + PBM and SER + PBM), showed a modulating effect on the inflammatory process of experimental second‐degree burns in animals, evidenced by the tendency to reduce the levels of some inflammatory markers, and by the deposition of extracellular matrix rich in collagen, suggesting an acceleration in the resolution of the lesion despite the absence of significant differences in the area of the lesion and temperature between the groups. The observed decrease in the antioxidant enzymes SOD and CAT may signal an early transition to the tissue maturation phase. However, some limitations were identified, including the inability to analyse the remodelling and resolution phases of wound healing at later time points, thereby precluding assessment of tensile strength and skin elasticity, as well as the final aesthetic appearance of the scar; therefore, further studies are warranted.

## Funding

This work was supported by Coordenação de Aperfeiçoamento de Pessoal de Nível Superior and Conselho Nacional de Desenvolvimento Científico e Tecnológico.

## Conflicts of Interest

The authors declare no conflicts of interest.

## Data Availability

The data that support the findings of this study are available on request from the corresponding author. The data are not publicly available due to privacy or ethical restrictions.
